# Theories of Fever

**Published:** 1879-10

**Authors:** E. P. Hurd

**Affiliations:** Newburyport, Massachusetts


### THEORIES OF FEVER.

By Dr. E. P. Hurd, of Newburyport, Massachusetts.

The hypothesis of calorific nerves and nervous centres (supported by Bernard) was discussed, and shown to be destitute of proof; that of a primitvie perturbation of the vaso-motor system was found to be equally wanting in solid foundation. In fevers the nervous symptoms are not constant, and when they occur they are secondary to the nutritive disorders. (The abnormal heat is not due to simple paralysis of the sympathetic, for division of the sympathetic does not produce fever.) The evidence all points to a pyretogenous cause at work in the blood, and by its irritating effects on the tissues, exaggerating all calorifacient chemico-vital processes. In short, the *humoral* theory is the best. The *materies morbi* of fever in general is unknown. The germ theory lacks inductive proof, and certainly cannot apply to sympathetic fever from wounds or surgical operations, etc., or to ephemeral or catarrhal fever. The hypothesis of a ferment in the blood, exciting and giving preponderance to disassimilation, is more probable; this ferment may be a chemical poison from the atmosphere, or it may be a morbid product of the system itself.

We are becoming more and more convinced that there is an orderly sequence of events in fevers as in all other phenomena of nature, there is law and not disorder even in disease, and that human skill and knowledge are impotent materially to modify the course of febrile diseases. There will be occasions when your frail bark will be tossed on angry billows, when by adroit manœuvres you may avoid rocks and quicksands; there will be other occasions when the utmost you can do is to determine your bearings, your latitude and longitude, powerless even to guide the craft amid the raging storm.

The treatment of fevers comprises the following principles: (1.) Eliminate the cause. (2.) Support the strength. (3.) Meet dangerous complications as they may arise. (4.) Rescue the organism from the baneful effects of the fever heat.

(1.) The first indication, to neutralize or remove the *mataries morbi*, cannot be efficiently met, because we do not know what the *materies morbi* is. It may be an altered condition of the blood from cold or heat, or constitutional cachexia, or from retained excreta, the pyretogenous substance acting as a ferment, poisoning the whole mass of the blood, and exciting to inordinate activity the organic combustions ; it may be a living germ from the vegetable world, or a degraded form of bioplasm.

We are wholly in the dark on this subject, and therefore cannot intelligently combat the *materies morbi.* We are certainly not warranted, on the basis of positive knowledge, in dosing our fever patients with antiseptics and antizymotics with the intent to neutralize in the blood or destroy the fever ferment or fever germ. I cannot except the traditional chlorate of potash and euchlorine, permanganate of potash and salicylic acid, carbolic acid and sulphocarbolate of sodium, sulphurous acid and bisulphite of sodium, or even, in this connection, quinine and alcohol. Until we have positive knowledge, a judicious and respectful skepticism is our highest wisdom. Nevertheless, while we may not aim our shaft at an imaginary foe, we do well to keep the emunctories open, as there is reason to believe that through the ordinary channels of excretion the fever poison passes out of the system. It is certain that return to health is coincident with return to normal activity of the organs of secretion and excretion. Hence the continued use of the customary sweet spirits of nitre finds justification; the vinum ipecacuanhæ in diaphoretic or expectorant doses; the acetate, citrate, and bicarbonate of patassa, and other mild diuretics; and the occasional laxative of senna, rhubarb, castor oil, or buckthorn when the bowels are confined.

My own limited experience does not lead me to repose much faith in aconite or other nerve sedatives as febrifuges.

It is very improbable that the morbid heat production is at all influenced by these drugs, or that they are in any marked degree antipyretic. (2.) The second indication, to support the vital forces, includes all foods and stimulants, as well as the hypnotics and anodynes which you give to procure sleep and relieve pain and restlessness. Doubtless an important advance in rational therapeutics has been made since bleeding and depressants in the treatment of fevers were abandoned, since Todd taught us to use alcholic stimulants more freely and Graves fed fevers. And yet just here caution and judgment are needed. I am convinced that many cases of continued fever do better without a drop of wine and only a moderate supply of liquid aliments. Others do better with a little wine or whiskey every two, three, or four hours, and an abundance of pure milk. Sometimes it is advantageous to begin the stimulant treatment early, as where the tendency to death is markedly by asthenia. We must combat the fever heat by our cold baths and quinine at the same time we stimulate with alcohol. Restlessness, wakefulness, and delirum must be controlled by camphor and Dover's powder, or better still with chloral, or the bromides with hydrobromic acid. (3.) The third indication, to meet complications as they may arise, comprehends all those measures, medical and surgical, necessary to arrest hæmorrhages, check diarrhœa, stay the progress of ulcerations, etc., attention to which is necessary to save the life of the patient. (4.) The fourth indication, which we can happily do much to fulfill, is to restrain as far as possible morbid heat production, or to save the tissues from its toxic effects.

Can anything be done to lesson heat production? Quinine in large doses is, I believe, the only safe antipyretic which even temporarily lessens organic combustions. Salicylic acid is of limited and doubtful utility. Clinical experience has determined that quinine is a veritable antipyretic, and therefore in a sense, specific in all fevers.

A few years ago we should have shuddered at the suggestion of giving to a child three years of age, laboring under a fever heat of 105°F., five grains of quinine every hour; with the view of bringing down the fever; now we find by experience that such doses produce no immediate bad effects, and that we can obtain a fall of several degrees by a few doses. Much larger doses may be given to adults, generally with gratifying results. Any cinchonism that ensues is of transient duration. The quinine in doses of a couple of grammes is often conjoined with the cold bath, with more marked antipyretic effect.

It is claimed that the antipyretic cuts short the febrile processes; the most that the advocates of this treatment claim is that by virtue of its anti-fermentative action on the blood or its tonic effect on the tissues, or by virtue of being a *germicide* quinine restrains excessive waste, promotes assimilation, checks the riotous production of bioplasm, and thus rescues the tissues, and especially the heart, from the destructive effects of high heat. If it acts as an antiseptic or germicide, it is certainly not very successful in its work, as it does not cut short the fever. To do good its use must be persevered in, and it must be given boldly. Whenever the temperature reaches 104° F. the quinine treatment must be commenced, and it must be given in repeated large doses at short intervals till the temperature falls nearly to the normal figure. (Ten grains an hour to an adult will bring down the fever heat after a few doses to nearly the normal.)

The next antipyretic to be mentioned, and probably the first in importance, is cold, applied in the form of cold baths, sponge baths, wrappings of ice-cold water, or ice-bags.

Twenty years ago it would have been considered madness to take a child, in the first stage of scarlet fever, manifesting delirum or stupor from febrile calorification and the force of the virus, immerse it in cold water, and keep it there for several minutes, pouring (it may be) cold water on the head of the child till rigor supervened, the thermometer indicating the point at which the child should be removed from the bath. Now this is done with seeming impunity, and is countenanced by good clinicians as legitimate practice. Some of us country physicians think that in desperate cases we have saved life by these means. In ordinary practice cold baths are inconvenient, and our patients are shy of this mode of treatment: cold sponging is much resorted to as a substitute. The patient is stripped of his clothing and laid on a rubber cloth; he is rapidly sponged from head to foot with ice-cold vinegar and water till the temperature falls from 104° or 105° to nearly 100° F.: then he is wrapped in a dry flannel blanket, and returned to his bed. The cold sponging is repeated whenever the thermometer indicates 104° F.

As to the results of the antipyretic treatment, after an experience of nine or ten years, we cannot speak very confidently. The immediate effects are generally very salutary, but the fever runs on; repeated baths somewhat exhaust the patient, and our large doses of quinine may do lasting harm. Certainly hospital statistics do not speak very encouragingly for the antipyretic treatment of fevers. But clinical statistics are notoriously unreliable. It is to such statistics that homœopaths appeal, and we know with how little reason. The antipyretic system seems to be theoretically sound, and we have probably yet to learn how it may be most safely and efficiently managed. We must feel our way along, proving all things and holding fast that which is good; we must persevere, hopeful; follow the best lights; where certainty is impossible be content to remain in doubt; indulge no vain dreams; obey the dictations of common sense.—*Boston Med. and Surg. Journal.*

